# People’s Knowledge and Attitudes About Factors That Can Impact Wound Healing in the Eastern Province, Saudi Arabia

**DOI:** 10.7759/cureus.50734

**Published:** 2023-12-18

**Authors:** Mohammed A Buhalim, Mohammed A Albesher, Maitham A Albesher, Nora J Alsultan, Haidar A Alessa, Farha A Aldossary

**Affiliations:** 1 General Surgery, King Faisal University, Al-Ahsa, SAU; 2 Medical School, King Faisal University, Al-Ahsa, SAU; 3 Medicine and Surgery, N.V. Sklifosovsky Institute of Clinical Medicine, Moscow, RUS

**Keywords:** wound care, healing delayed, knowledge and attitude, wound, ­wound healing

## Abstract

Background

Wounds, ranging from acute to chronic, demand timely intervention to prevent complications. Infections can impede healing, leading to sepsis. Chronic wounds impact society, causing limitations in mobility and social exclusion. Early identification of infections is crucial for effective treatment, reducing unnecessary antibiotic use, and improving patient outcomes.

Methodology

This cross-sectional study was conducted from June to October 2023 in Saudi Arabia using a self-administered online questionnaire to assess wound healing knowledge and attitudes. Convenient random sampling via social media was employed to collect data. Data analysis was performed using SPSS version 23 (IBM Corp., Armonk, NY, USA).

Results

Our study predominantly featured female (67.1%), Saudi (94.8%), aged 18-25 years (42.0%), and married (51.1%) participants. Chronic illnesses were prevalent in 24.2%, with heart disease (5.7%) and hypertension (4.5%) being notable. Burn injuries (27.4%) and upper extremity wounds (40.9%) were common, prompting immediate medical care (54.1%). Internet sources (22.6%) and relatives/friends (18.4%) were key for wound care information. Participants displayed solid awareness of wound-related factors, with notable misconceptions regarding Zamzam water (44.4%) and coffee beans (39.3%). Participants prioritized stopping bleeding (41.1%) and using wound patches (42.1%) for home injuries. Age group, education, marital status, and occupation impacted knowledge about wound healing.

Conclusions

Our study highlights key demographics, prevalent chronic illnesses, common wound types, and crucial sources of wound care information. Participants’ awareness, coupled with notable misconceptions, emphasizes the importance of tailored education. Factors such as age, education, marital status, and occupation impact knowledge about wound healing.

## Introduction

A wound may be defined as any disruption of the integrity of skin, mucous membrane, or organ tissue. Thus, skin functionality is affected as a result of the disruption of cellular and anatomical structures [1]. Skin breaks can be caused by several factors, including illnesses, injuries, or burns [2]. There are various types of wounds, ranging from minor to severe, and the type of treatment required depends on the severity of the wound. An acute wound is caused by surgery or burns, whereas a chronic wound has other causes, including leg ulcers, diabetic foot ulcers, and pressure ulcers [2]. Acute wounds need specialized care, whereas chronic wounds may require a longer-term approach to heal. Additionally, it could be defined as a non-healing wound for more than six weeks. To manage wounds successfully, early intervention is imperative [3]. Globally, 50 million people have been injured by wounds since 2015 [4]. Approximately 6 million lacerations are treated every year in American emergency departments (EDs) [5]. Studies indicate that over 5 million people suffer from chronic wounds that require medication and management due to car accidents and systemic diseases, particularly diabetes [6]. Due to wound infection, wound healing may be slowed, and, in the worst-case scenario, an individual may develop sepsis, which can be fatal [7]. Moreover, chronic wounds are associated with hidden tensions in the community as a result of limitations in mobility, limited functional abilities, social isolation, loss of labor market participation, and social exclusion [8]. The introduction of care in conjunction with evidence-based guidelines has resulted in reported improvements in healing and recurrence rates [8]. An individual’s health can be adversely affected by improper wound treatment [3]. A wound that is not adequately treated can lead to infection (3%-15%) [9,10]. Fever, swelling, pain, and purulent exudate are common symptoms of wound infection [11]. It is important to note that certain factors, such as corticosteroid use, smoking, and poor general health conditions, impair wound healing [12]. A poor wound care regimen and neglect of the consequences of inadequate wound care cause a tremendous financial burden and a significant reduction in the quality of life for patients [13]. It is vital to identify wound infections early and accurately initiate appropriate treatment in a timely manner and to prevent further complications. As a result of accurate diagnosis of infection, unnecessary antibiotic use can be avoided [7]. In this study, we aim to assess the knowledge and attitude toward wound healing and the factors that can affect it.

## Materials and methods

Study methodology

This cross-sectional study targeted the general population of Eastern Province, Saudi Arabia to determine the knowledge and attitudes about the factors that impact wound healing. The study was conducted between June 2023 and October 2023. An online survey was created using Google Forms for data collection. The online survey was disseminated to the general population in Eastern Province, Saudi Arabia, and people were encouraged and invited to participate. To enroll as many participants as possible, two or more data collectors were recruited from every city. Data were collected through an online self-administered questionnaire where participants first consented to participate in the study before filling out the questionnaire. The questionnaire included three sections, of which the first section asked about sociodemographic data, including gender, nationality, education level, habit of smoking and any comorbidities, occupation, and marital status. The second part asked about general characteristics such as the type, site, source, signs, and symptoms of wound care. The third section asked about various factors affecting wound care by asking questions with three options of “agree,” “disagree,” and “don’t know.” The questionnaire we utilized was partly taken from a different study and modified to fit our practices and culture in addition to other studies [1,3]. The study was presented to specialists in general surgery for improvement and approval. It was first developed in English and then translated into Arabic to be comprehensible for the targeted population. The Arabic version was examined by three language experts, and the translation was approved after grammatical and linguistic modifications. After that, a pilot study was performed on a small group of people (15 persons) to confirm a uniform understanding of the questions. Ethical approval was obtained from the ethics committee of King Faisal University (approval number: ETHICS1227).

Study population

The study subjects were the entire general population of Eastern Province, Saudi Arabia who consented to participate in this study between June and October 2023 and met the inclusion criteria. The inclusion criteria consisted of being an adult aged 18 years and older; living in Eastern Province, Saudi Arabia; and consenting to participate in the study. The exclusion criteria consisted of being younger than 18 years old; living outside of Eastern Province, Saudi Arabia; and not consenting to participate in the study. The sampling technique utilized was convenient random sampling where the questionnaire was disseminated via social media platforms, and the general population of Saudi Arabia was invited to participate through an online link. The sample size was calculated using the formula n = z2pq/d2, with a confidence level of 95%, an estimated proportion of 50%, and a 5% level of precision. The minimum sample size was calculated to be 385. However, more participants and candidates were included to ensure sufficiency.

Study procedure

According to the inclusion and exclusion criteria, certain participants who fulfilled the criteria and agreed to the given consent were enrolled. Each participant anonymously filled out the questionnaire. The results of the questionnaires were analyzed statistically. The results of the study were interpreted accordingly. Interpretation of the collected data was done accordingly, and proposals for potential solutions, when applicable, were delivered.

Data management

The data were stored in a trusted place, and only approved personnel had access to the data. Privacy and confidentiality were a priority, there was no part of this study threatening participants’ confidentiality, and the identities of the participants were unknown. The data were analyzed and interpreted later by the investigator.

Statistical analysis

Data analysis was performed using SPSS version 23 (IBM Corp., Armonk, NY, USA). Frequency and percentages were used to present categorical variables. Minimum, maximum, mean, and standard deviation were used to present numerical variables. The chi-square test was used for comparison between variables. The level of significance was set at 0.05.

## Results

Our study included 1,522 participants from the Eastern Province of Saudi Arabia. Table 1 shows that the majority were females (1,021, 67.1%), Saudis (1,443, 94.8%), and aged 18-25 years (639, 42.0%). Participants were mostly married (777, 51.1%) and had a bachelor’s education (545, 35.8%). Most participants were students by occupation (519, 34.1%) and earned less than 5,000 SAR monthly (775, 50.9%). Most participants were non-smokers (1,340, 88.0%).

**Table 1:** Sociodemographic and other parameters of participants.

		Frequency (n = 1,522)	Percentage
Gender	Females	1,021	67.1
	Males	501	32.9
Age	18–25 years	639	42.0
	26–40 years	382	25.1
	41–60 years	457	30.0
	>60 years	44	2.9
Nationality	Non-Saudi	79	5.2
	Saudi	1,443	94.8
Marital status	Single	683	44.9
	Married	777	51.1
	Separate	41	2.7
	Widowed	21	1.4
Educational status	Higher school or less	286	18.8
	College education	455	29.9
	Bachelors	545	35.8
	Diploma	167	11.0
	Masters or higher	69	4.5
Occupation	Student	519	34.1
	Work in non-health sector	338	22.2
	Housewife	216	14.2
	Work in health sector	151	9.9
	Retired	114	7.5
	Unemployed	86	5.7
	Other	98	6.4
Monthly income	<5,000 SAR	775	50.9
	5,000–10,000 SAR	317	20.8
	>10,000 SAR	430	28.3
Smoking status	Non-smokers	1,340	88.0
	Smokers	156	10.2
	Ex-smokers	26	1.7

**Figure 1:**
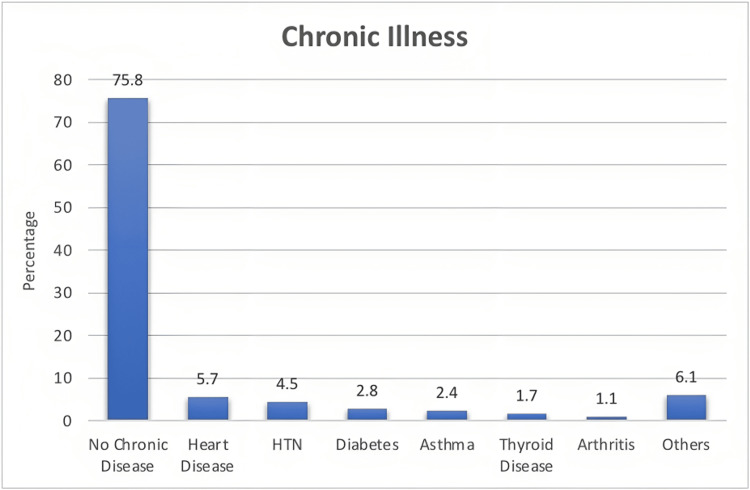
Different chronic illnesses in participants. HTN: hypertension

Figure 1 shows the prevalence of chronic illnesses among participants from the Eastern Province of Saudi Arabia. The majority had no chronic diseases (1,153, 75.8%), with heart disease (86, 5.7%) and hypertension (68, 4.5%) being the most prevalent. Other chronic illnesses accounted for the remaining percentages.

Table 2 shows the characteristics of wounds among participants, who reported various previous wound experiences, with burns (418, 27.4%) and injuries (383, 25.1%) being the most common. Most wounds were located on the upper extremities (624, 40.9%). Most sought medical care immediately (824, 54.1%). Seeking medical advice was primarily prompted by wide/deep wounds (1,164, 76.4%), severe bleeding (1,150, 75.5%), head injury (1,016, 66.7%), and the appearance of infection symptoms (1,003, 65.9%).

**Table 2:** Different characteristics of wounds documented by the participants.

		Frequency (n = 1,522)	Percentage
Previous experience of wound care for what type of wounds	No wound	485	31.8
	Burn	418	27.4
	Injury	383	25.1
	Wound	372	24.4
	Surgical incision	194	12.7
	Episiotomy	93	6.1
	Ulcer	86	5.6
	Accident	79	5.1
	Diabetic wound	20	1.3
	Cut	10	0.6
Location of wound	Upper extremities	624	40.9
	Never been cut	438	28.7
	Feet	263	17.2
	Leg	174	11.4
	Face	171	11.2
	Abdomen	146	9.5
	Pelvis	99	6.5
	Thigh	99	6.5
	Neck	52	3.4
	Chest	45	2.9
	Back	15	0.9
Time between injury and initial medical care	Medical attention is not needed	469	30.8
	Immediately	824	54.1
	Within 1–3 days	136	8.9
	Within 4–7 days	40	2.6
	>1 week	53	3.5
Seek medical advice due to following signs/symptoms	No	178	11.6
	Wide/Deep wound	1,164	76.4
	Severe bleeding	1,150	75.5
	Head injury	1,016	66.7
	Infection symptoms appear	1,003	65.9
	Foreign body in the wound site	914	60.1
	Bite/Injury from dirty/rusty objects	861	56.5
	Heavy bleeding	34	2.2

**Figure 2:**
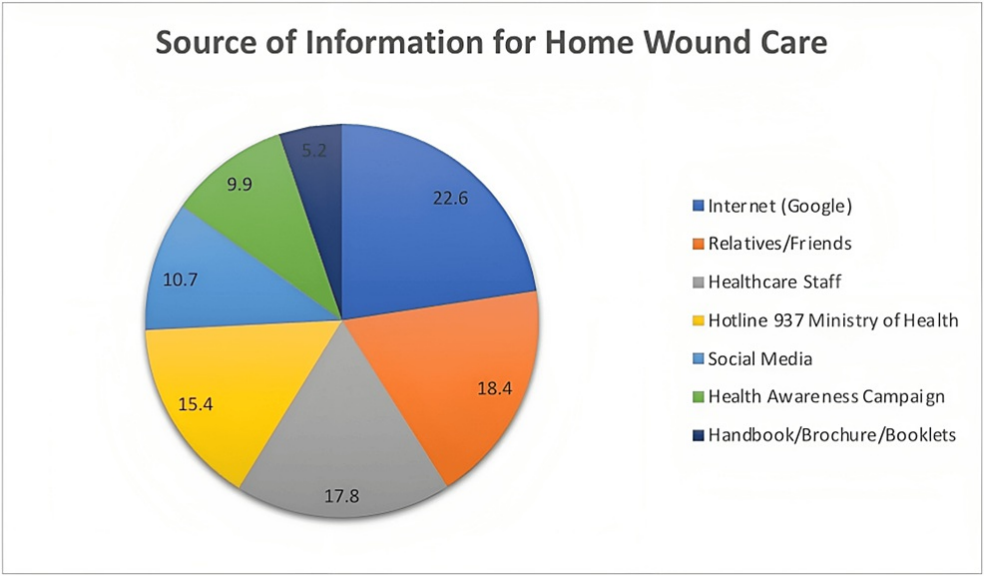
Different information sources for home care of wounds.

Figure 2 shows that the majority of participants obtained information for home wound care from the Internet (343, 22.6%) and relatives/friends (280, 18.4%). Healthcare staff (270, 17.8%) and the Ministry of Health’s hotline 937 Ministry of Health (234, 15.4%) also served as significant sources. Social media (162, 10.7%)and health awareness campaigns (150, 9.9%) were also utilized, albeit to a lesser extent.

Table 3 shows people’s knowledge and attitudes concerning factors that can impact wound healing. The majority of participants recognized the significance of good nutrition for wound healing (1,465, 96.3%) and the necessity of handwashing before changing wound dressing (1,497, 98.4%). Moreover, most participants were aware that unpleasant smells in wounds are caused by a bacterial infection (1,435, 94.3%). Additionally, a considerable proportion acknowledged that diabetes does not delay wound healing (333, 21.9%), the negative impact of smoking on wound healing (1,267, 83.2%), and the necessity of antibiotics for wound healing (945, 62.1%). There was a recognition that wound care is typically performed by someone other than the injured person (1,163, 76.4%). There was a perception that topical honey use is effective in wound healing (629, 41.3%) and that bathing may delay wound healing (724, 47.6%). Moreover, a significant majority believed that exposing wounds to fresh air promotes wound healing (1,052, 69.1%), while others acknowledged that obesity can delay wound healing (856, 56.2%). Additionally, participants recognized the potential effects of cortisone on healing time (1,086, 71.4%) and the role of age in wound healing (1,336, 87.8%). However, there were misconceptions, such as the belief in the beneficial effects of using Zamzam water to improve healing time (676, 44.4%) and the anti-inflammatory effect of saltwater (1,029, 67.6%). Moreover, participants acknowledged the anti-inflammatory effect of topical aloe vera application (880, 57.8%) and the potential utility of vaseline in reducing wound scars (769, 50.5%). Additionally, the majority recognized the importance of vitamins C, A, K, zinc, and magnesium in the wound healing process (1,389, 91.3%). However, there seemed to be a misconception about the usefulness of coffee beans in controlling bleeding (598, 39.3%). Furthermore, whereas most participants understood that the location, size, or shape of the wound can impact healing time (1,142, 75.0%), a substantial proportion held the opposite belief (380, 25.0%).

**Table 3:** Peoples’ knowledge and attitude about factors that can impact wound healing.

		Frequency (n = 1,522)	Percentage
Good nutrition is essential for wound healing	No	57	3.7
	Yes	1,465	96.3
Hand washing is needed before changing wound dressing	No	25	1.6
	Yes	1,497	98.4
Unpleasant smells in the wound are caused by bacterial infections	No	87	5.7
	Yes	1,435	94.3
Diabetes does not affect delayed wound healing	No	1,189	78.1
	Yes	333	21.9
Smoking negatively affects wound healing	No	255	16.8
	Yes	1,267	83.2
Antibiotics are necessary for wound healing	No	577	37.9
	Yes	945	62.1
Wound care is performed by other than the injured person	No	359	23.6
	Yes	1,163	76.4
Topical use of honey helps in wound healing	No	893	58.7
	Yes	629	41.3
Bathing may delay wound healing	No	724	47.6
	Yes	798	52.4
Exposing wounds to fresh air promotes wound healing	No	470	30.9
	Yes	1,052	69.1
Obesity delays wound healing	No	666	43.8
	Yes	856	56.2
Using Zamzam to wash wounds improves healing time	No	846	55.6
	Yes	676	44.4
Cortisone may affect healing time	No	436	28.6
	Yes	1,086	71.4
Saltwater has an anti-inflammatory effect	No	493	32.4
	Yes	1,029	67.6
Age plays an important in wound healing	No	186	12.2
	Yes	1,336	87.8
Topical application of home remedy (aloe vera) has an anti-inflammatory effect	No	642	42.2
	Yes	880	57.8
Vaseline is useful for reducing wound scar	No	753	49.5
	Yes	769	50.5
Vitamins C, A, K, zinc, and Mg play an important role in the wound healing process	No	133	8.7
	Yes	1,389	91.3
Coffee beans are useful in controlling bleeding	No	924	60.7
	Yes	598	39.3
Location, size, or shape of the wound has no role in healing time	No	1,142	75.0
	Yes	380	25.0

Table 4 reveals participants’ practices in case of home injuries. Most participants would prioritize stopping bleeding (624, 41.1%) and would utilize wound patches (641, 42.1%) as the preferred type of bandage. Sterilizing wipes (1,344, 88.3%) were predominantly chosen as the solution for cleaning wounds, reflecting a commitment to immediate and effective wound management practices at home.

**Table 4:** Participants’ practices in case of injury at home.

		Frequency (n = 1,522)	Percentage
What you will do in case of an injury at home	Stop bleeding	624	41.1
	Cover the wound and go to the hospital	443	29.1
	Clean the wound	339	22.3
	Go to the hospital	72	4.7
	Apply antibiotic ointment and go to hospital	44	2.9
Type of bandage used at home	Wound patch	641	42.1
	Dry gauze	463	30.4
	Cotton	237	15.5
	Wet gauze	83	5.4
	Towel	60	3.9
	Depending on wound	9	0.5
	Napkin	8	0.5
	All of the above	15	0.9
	Other	13	0.8
Solution used to clean wounds at home	Sterilizing wipe	1,344	88.3
	Tap water	446	29.3
	Mercurochrome	346	22.7
	Betadine	303	19.9
	Baby wipes	152	9.9
	Perfume	96	6.3
	Hydrogen peroxide	55	3.6
	Sodium chloride	7	0.4
	Dettol	4	0.2

Table 5 shows the association between participants’ understanding of factors influencing wound healing and various sociodemographic factors. Significantly higher knowledge was observed among the 18-25, 26- 40, and 41-60 age groups (p < 0.001). Similarly, bachelor’s education and student by occupation were associated with superior knowledge (p = 0.011 and 0.003, respectively). Marital status also exhibited a significant association (p = 0.007), with married individuals having more knowledge. Gender, nationality, monthly income, and smoking status did not demonstrate significant correlations with knowledge levels (p > 0.05).

**Table 5:** Association of knowledge about factors affecting wound healing and different sociodemographic factors.

		Knowledge about factors affecting wound healing		P-value
		Poor knowledge	High knowledge	
Age	18–25 years	240	399	<0.001
	26–40 years	123	259	
	41–60 years	117	340	
	>60 years	11	33	
Gender	Female	337	684	0.374
	Male	154	347	
Nationality	Non-Saudi	25	54	0.904
	Saudi	466	977	
Marital status	Single	244	439	0.007
	Married	236	541	
	Separate	9	32	
	Widowed	2	19	
Educational level	Higher school or less	83	203	0.011
	College education	164	291	
	Bachelors	182	363	
	Diploma	37	130	
	Masters or higher	25	44	
Occupation	Student	194	325	0.003
	Work in the non-health sector	98	240	
	Housewife	55	161	
	Work in the health sector	59	92	
	Retired	29	85	
	Unemployed	30	56	
	Other	26	72	
Monthly income	<5,000 SAR	256	519	0.777
	5,000–10,000 SAR	98	219	
	>10,000 SAR	137	293	
Smoking status	Non-smokers	442	898	0.260
	Smokers	42	114	
	Ex-smokers	7	19	

## Discussion

Wounds, if untreated, pose serious risks, necessitating tailored management for acute and chronic cases. Infections and subsequent complications, such as sepsis, underscore the importance of timely intervention and adherence to evidence-based protocols. Patient education about proper wound care is critical due to the potential for severe complications. Recognizing factors that impede healing is crucial for effective treatment and improved patient outcomes. Our study provides a comprehensive understanding of wound healing in the Eastern Province of Saudi Arabia, encompassing participant demographics, chronic illness prevalence, wound characteristics, sources of wound care information, knowledge and attitudes about wound healing, practices for home injuries, and the associations between wound healing knowledge and sociodemographic factors.

The study’s demographic findings indicate a prevalence of females, young adults, and married individuals, mirroring the local population profile in Saudi Arabia. Similarly, the dominance of participants with bachelor’s degrees and lower income aligns with the region’s typical educational and economic patterns.

The prevalence of chronic diseases, particularly heart disease and hypertension, among the participants underscores the importance of addressing the specific challenges these individuals may face in wound healing. These findings are consistent with the previous studies which showed that heart diseases impaired the healing process in obese patients and hypertensive patients had a higher risk of prolonged wound discharge after total hip arthroplasty than their normotensive counterparts [12,13]. Understanding the impact of these comorbidities on wound healing can guide healthcare professionals in implementing targeted interventions to improve healing outcomes.

Regarding the characteristics of wounds and medical care-seeking behavior, there is a high prevalence of burns and injuries, especially on the upper extremities, which emphasizes the significance of providing specialized wound care tailored to the nature and location of these wounds. The prompt seeking of medical attention, primarily driven by specific wound characteristics, indicates the community’s awareness of the importance of immediate and appropriate medical care for effective wound management [14].

The reliance on the Internet and healthcare professionals as primary sources of wound care information indicates the growing influence of technology and medical expertise in disseminating knowledge about wound care. Welsh et al. (2018) showed that the hospital’s wound care specialist nurses are the primary source of information for participants to manage acute wounds [15]. This highlights the potential for utilizing these platforms to implement targeted health education programs and ensure the accurate dissemination of wound care information among the community.

Regarding the knowledge and attitudes about wound healing, there is a high level of awareness regarding the importance of good nutrition, handwashing (prevents infections and promotes healing), and the detrimental effects of smoking on wound healing, which reflects a commendable understanding of basic wound care principles among the participants [16-18]. However, the prevalence of certain misconceptions, such as the belief in the anti-inflammatory effect of saltwater and the utility of coffee beans in controlling bleeding, suggests the need for targeted educational initiatives to address these misunderstandings and promote evidence-based wound care practices.

Regarding the practices in case of home injuries, the prioritization of stopping bleeding and the preference for using sterilizing wipes for wound cleaning among participants indicate a strong commitment to immediate and effective wound management at home. Atiyeh et al. (2009) showed that sterile wound dressing prevents infection and may be used in the irrigation process [19]. These practices align with the recommended first aid measures and underscore the community’s preparedness to address common injuries promptly.

Finally, we observed the associations between wound healing knowledge and sociodemographic factors, including age, education, occupation, and marital status, emphasizing the potential influence of these factors on individuals’ understanding of wound care [20]. These findings underscore the importance of tailoring educational interventions to bridge knowledge gaps and promote uniform awareness of wound care practices across different demographic groups.

Comparatively, our findings corroborate with previous research, indicating a general understanding of basic wound care principles among the population. However, certain unique trends, such as the prevalent use of the internet for wound care information and the persistence of specific misconceptions, underscore the need for targeted educational interventions to address the specific challenges identified in the Eastern Province context. Additionally, our study contributes valuable insights into the community’s practices and perceptions related to wound care, further enriching the existing literature on wound healing practices in the region.

Limitations

Several limitations of the study include potential recall bias due to self-reported data, which could affect the accuracy of participant responses. Additionally, the cross-sectional design may limit the establishment of causal relationships, warranting further longitudinal investigations for comprehensive understanding.

## Conclusions

Our study provides comprehensive insights into the knowledge, attitudes, and practices concerning wound healing among the population of the Eastern Province of Saudi Arabia. The findings underscore the need for targeted educational interventions, the promotion of evidence-based wound care practices, and the dissemination of accurate wound care information to foster optimal wound healing outcomes in the community. Further research is essential to explore specific strategies for addressing the identified knowledge gaps and improving wound care practices in the region.
